# How photoautotrophy, photomixotrophy, and ventilation affect the stomata and fluorescence emission of pistachios rootstock?

**DOI:** 10.1515/biol-2021-0115

**Published:** 2021-10-21

**Authors:** Mohammad Javad Mahmoudi Meimand, Mohammad Hossein Shamshiri, Khalil Malekzadeh, Mohammad Reza Dehghani

**Affiliations:** Department of Horticulture, Faculty of Agriculture, Vali-e-Asr University of Rafsanjan, Rafsanjan, Iran; Department of Genetics and Plant Production, Faculty of Agriculture, Vali-e-Asr University of Rafsanjan, Rafsanjan, Iran; Department of Biotechnology and Plant Breeding, Faculty of Agriculture, Vali-e-Asr University of Rafsanjan, Rafsanjan, Iran

**Keywords:** chlorophyll, photosynthesis, acclimatization, desiccation, *F*_v_/*F*_m_

## Abstract

The effects of ventilation and sucrose concentration on proliferation and organogenesis of pistachio cutting and photosynthetic performance of two *in vitro* cultures of pistachio rootstocks have been assessed. The apical leaf buds (Qazvini and UCB1 cultivars) were cultured in filter vessels containing Murashige and Skoog medium supplemented with 0, 10, 15, and 30 g L^*−*1^ of sucrose. The plants treated with 10, 15, and 30 g L^*−*1^ sucrose showed no significant differences regarding the measured traits; therefore, this treatment was set aside from the final statistical analyses. Use of different ventilation systems showed to be suitable for increasing the growth of pistachio. Referring to root production difficulties under *in vitro* cultivation of pistachio, ventilation increased the root production and length. However, the full ventilation system was more effective in improving the growth properties. Regression between fluorescence feature vs root length showed that *F*_v_/*F*_m_ had a significant positive relationship with root length. Stomata of cell parameters under ventilation systems improved compared to no ventilation, which was highly similar to the trend in the greenhouse. The overall results indicated that low concentrations of sucrose (e.g., 10 g L^*−*1^) and full ventilation are recommended for producing high quality and vigorous pistachio plantlets under *in vitro* conditions.

## 1 Introduction

Perennial trees, such as pistachio (*Pistacia vera*), require a long time to go from cutting to a plant with root and shoot. In pistachio, the average time from the cutting to a tree that can produce the nuts is about 2–3 years. In addition, the diseases and some other problems such as insect attach and fungi infections would threaten the maternal plants under *in vivo* conditions. Therefore, using tissue culture for producing such plants can shorten the production period from the cutting to tree in comparison with *in vivo* conditions; in pistachio, from 3 years to 1 years, it can also provide new plants without any infection [1]. The maximum time required for transforming the produced plants from acclimatization to the point when the transported plant starts to continue its normal growth is 1 year, whereas its final nut yield is not significantly different from *in vivo* produced trees in the following years. Furthermore, the breeding of perennial trees has always been hard to improve, whereas using tissue culture would provide much more opportunity to improve the quality and quantity of these plants. In tissue culture, a researcher can assess the impacts of different substances with mutation effects and other ways to test and improve the quality of perennial trees. However, cultivating pistachio has had some essential improvement since its first achievement in growing under the *in vitro* condition as rooting and root genesis of the tissues in any environment and medium have faced some difficulties [2]. Successful rooting in pistachio trees under *in vitro* conditions, or any other provided condition to grow vigorous root, would improve its growth and the economic production of pistachio. Aside from improving the organogenesis of pistachio under *in vitro* condition, producing healthy and high-quality plants in high quantities is also among the main goals of *in vitro* production of pistachio. By providing and producing healthy and vigorous plants under *in vitro* conditions, the adaptability of such plants would successfully increase and allow them to have a higher chance to stand along under outdoor conditions [1]. One of the most important features for improving the quality and quantity of plants is to increase the rate of photosynthesis in plants in any possible way. Under *in vitro* conditions, the air usually is not substitutable. The content of oxygen and carbon dioxide is limited; accordingly, inventing some safe ways to circulate the air in the culture containers would probably improve plant production efficiency and produce high-quality plants. In addition, increasing the photosynthesis efficiency and the ability to use air and light efficiently have not gained enough interest in pistachio, and it practically has not shown any progress; therefore, pistachio requires extra consideration regarding the photosynthesis and improving its mechanism. Moreover, the chlorophyll (Chl) fluorescence emission assessment has been a helpful method for monitoring the plants’ photosynthesis ability and for determining the relative effects of different stress conditions on the plants [3].

In outdoor conditions, there is no limitation related to carbon dioxide and oxygen sources for being provided by the environments to the pistachio plants; whereas, under *in vitro* conditions, there are limitations for the amount of these sources for the plants. The amount of these sources is strictly dependent on and limited to their container volume. Meanwhile, ventilation can create better conditions for plantlets’ growth by increasing both the waxy leaf layer and stomatal functions [4]. Previous references have indicated that the thin leaf waxy layer and disordered stomatal cell function, especially open stomata, could lead to weak transpiration adjustment on the *in vitro*-grown plants [5]. Similarly, stomatal density seems to be a major adaptive trait in tissue-cultured plants [6], so that low stomatal density has been considered a critical determinant for high-efficient water use [7]. A similar report showed that stomatal density can tightly control plant water loss through the plant leaf surface and their closing mechanism in environmentally abnormal conditions, especially in tissue-cultured plants [8]. It has been also demonstrated that reducing stomatal density could increase the water-use efficiency [6]. Other stomatal traits, such as stomatal density, the stomatal index, epidermal cell density, and stomatal width, are required to be considered in tissue-cultured plants under different provided conditions and media to set up the best combinations for improving the quality of such traits in pistachio plants produced under *in vitro* conditions [9].

To the best of our knowledge, no studies on the effect of ventilation on the pistachio plants and their photosynthetic performance under these conditions were performed. Therefore, the main objectives of this study were to focus on the effect of ventilation mechanism on quality and quantity of pistachio plantlets growing under *in vitro* condition and to study the stomata anatomy and fluorescence emission under different ventilations interacting with sucrose treatment as the most important source of energy in *in vitro* plants.

## 2 Materials and methods

### 2.1 Plant material

Pistachio apical leaf buds from two rootstocks (Qazvini and UCB1) were cultured in vessels containing modified MS media optimized for both rootstocks and supplemented with 6-benzyladenine (BA; 1.5 mg L^−1^) and indole-3-butyric acid (0.1 mg L^−1^), which solidified with agar (7 g L^−1^). The sucrose source was mixed with media before its solidification by the rate of 0, 10, 15, and 30 g L^*−*1^ as the sucrose treatments. The media pH was adjusted to 5.7 before mixing the applied substances and autoclaving (20 min at 121°C) the liquid from media. UCB1 rootstock as a vigorous hybrid rootstock and Qazvini as a native stock were selected for this experiment because they are both highly tolerant of harsh conditions, especially salty and dry soil.

To assess the effect of ventilation, changeable vessel filters made out of 50 µm microporous polypropylene membrane (Pardis Co, Iran^®^) were applied. Accordingly, after mixing with agarose, the liquid medium was immediately poured into the bottom of the vessels, where the cuttings were planted. The neck of the vessels was connected with three different ventilation conditions, including full ventilation (FV), half ventilation (HV), and without ventilation (NV). In the vessels’ neck, two filters were placed at 5 cm distance from each other. The ventilation systems obtained the air from a container that had two fans for inserting the air. The air in the air container was properly disinfected by using UV light. The used vessels had one input path connected with the air container and contained two extra filters to insert the bottom of the vessels directly on the cutting and output path for exiting the air. At the end of the vessels’ output path, some mechanisms were used to ensure that air could not get in. Eight suction engines were used for inserting the air from the air container, and each output part of these engines was divided between ten ventilator systems. In FV systems, the period of 8 h out of 16 light hours (16/8 light–dark condition in the growth chamber) was used for inserting the air, but for HV, the period of 4 h was used. After each inserting period, the output and input parts of the vessels were sealed until the nest ventilation period. To be able to compare the ventilation treatments with *in vivo* control conditions, some pistachio cuttings were grown under greenhouse conditions (greenhouse plants [GP]). For each treatment combination, three ventilation vessels were used as repetitions, and the three separate cuttings were placed in each vessel’s bottom. Accordingly, the overall experimental units were equal to 72 (four sucrose levels × three ventilations × two rootstock × three repeats). The experimental units, alongside the air container, were placed in a growth chamber having 25 ± 2°C temperature and 16/8 h light–dark cycles. In addition, the control plants in the greenhouse condition were repeated three times and cultivated in pots with 500 mL volume, where each pot consisted of two cultivate cuttings.

### 2.2 Plantlet growth parameters

Forty-five days after starting the experiment, one of the three planting plantlets in each experimental unit was carefully taken out for measuring plantlet height and root length by using digital collis (caliper) according to the millimeter unit. Other growth parameters contained the number of shoots (proliferation) and leaves that were measured from all three plantlets in each experimental unit and used their average as the final result. Similar measurements were performed on GP.

### 2.3 Fluorescence emission assessment

**Figure 1:**
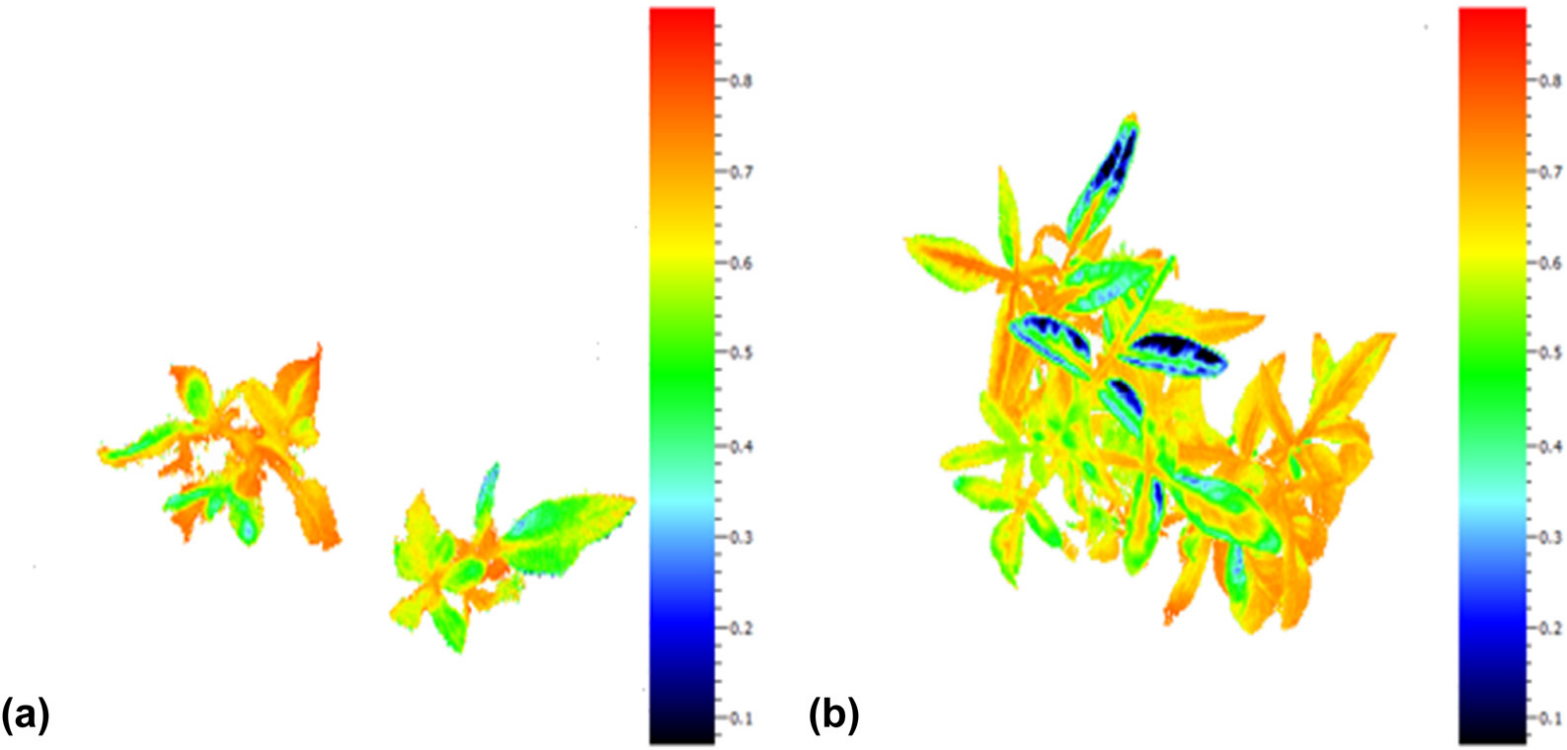
Fluorescence emission from two pistachio rootstocks UCB1 (a) and Qazvini (b) leaves. Vertical values are *F*_v_/*F*_m_ amounts for each rootstock, yellow-to-red-colored sections parts, maximum *F*_v_/*F*_m_ amounts.

For analyzing fluorescence emission, fully developed leaves were used for surveying the maximum quantum efficiency of Photosystem II (*F*_v_/*F*_m_). First, vessels were inserted in dark condition for at least 20 min to adapt to the dark. Next, plantlets were used to measure Chl fluorescence of *in vitro* plants and GP using a fluorometer system (Handy FluorCam FC 1000-H Photon Systems Instruments, PSI, the Czech Republic^®^) immediately. Images taken by FluorCam were recorded during short measuring flashes in dark conditions. Then, based on the FluorCam protocol, the leaf samples were then exposed to a pulse of saturating light (3,900 μmol m^−2^ s^−1^), resulting in a temporary photochemistry saturation and primary Quinone acceptor reduction of PSII [3,4]. After reaching a steady state of fluorescence, two main fluorescence data involving *F*_0_ and *F*_m_ were digitalized, during short measuring flashes in the dark (*F*_0_), and during the saturating exposition (*F*_m_) along with *F*_m′_ were obtained. Later, according to the ratio between *F*_m_ and *F*_0_, the variable fluorescence (*F*_v_) was calculated. Finally, the *F*_v_/*F*_m_ ratio and nonphotochemical chlorophyll fluorescence quenching (NPQ) were estimated according to the *F*_m_/*F*_m_ ratio [10] (Figure 1).

### 2.4 Stomatal studding

**Figure 2:**
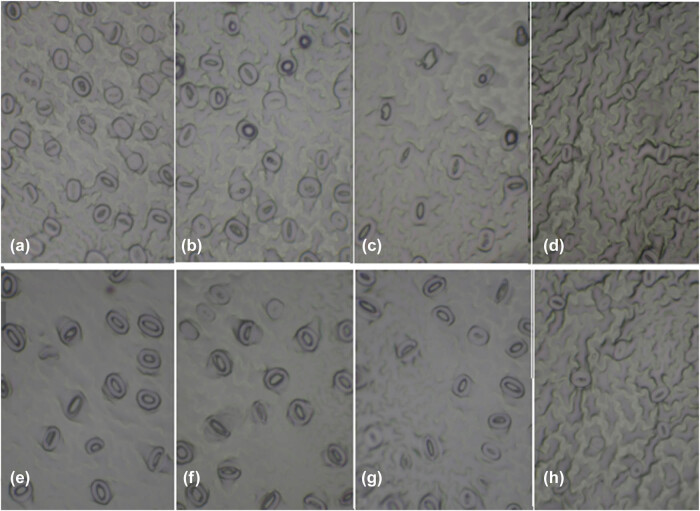
The shape of stomatal and epidermal cells of pistachio rootstocks Qazvini and UCB1, (a) UCB1 no ventilation, (b) UCB1 HV, (c) UCB1 FV, (d) UCB1 greenhouse plant, (e) Qazvini no ventilation, (f) Qazvini HV, (g) Qazvini FV, and (h) Qazvini greenhouse plant.

To analyze stomatal of the tissue-cultured plants, leaf samples were taken from samples supplied with sucrose 15 (g L^−1^) for all ventilation treatments. Next, based on a procedure described in a study [9], four-leaf samples from greenhouse pistachio plants and four vessels with two explants per vessel (as *in vitro* pistachio plantlets) were used for comparison. Furthermore, we used the nail varnish method for imaging the stomata cell, as explained in a previous study [10]. It should also be noted that plants were randomly selected from each replicate. Afterward, a thin layer of nail polish was applied to the abaxial surface of the second leaf of each sample. After 5 min, the dried varnish was gently peeled off, and the lower surface of the leaf epidermis was removed and placed on a lam. Finally, images of epidermal strips were taken (Figure 2) with a stereo microscope (SZM-3 model, Italy^®^). These images were used for calculating density (no mm^−2^), dimension (width and length [µm]), width-to-length ratio of stomata, as well as the stomatal index using Image Tools (University of Texas, TX), and the following equation was reported according to an earlier study [11]:

\begin{array}{c}\text{Stomatal}\hspace{.25em}\text{index}\hspace{1em}=\hspace{.25em}(\text{Stomatal}\hspace{.25em}\text{density}\times 100)/(\text{Stomatal}\hspace{.25em}\text{density}\\ \hspace{1em}+\text{Density}\hspace{.25em}\text{of}\hspace{.25em}\text{subsidiary}\hspace{.25em}\text{and}\hspace{.25em}\text{epidermal}\hspace{.25em}\text{cells})\end{array}]

### 2.5 Stomatal response to desiccation

Leaf relative water content (RWC) was calculated as a percentage during the desiccation period according to an earlier study [9]. To do so, first, the petioles were removed, and the fresh weight of pistachio leaf samples from greenhouse plants as well as *in vitro* plants was calculated in an environment with 50% relative humidity (RH) and temperature of 21°C resulted in 1.24 kPa vapor pressure deficit (VPD) and 50 μmol m^−2^ s^−1^ irradiance. Next, the leaves were dipped in distilled water for 24 h, and their turgid weight (TW) was measured. The procedure was continued with the gravimetrical weighing of TW every 5 min within 60 min. After desiccation, leaves were dried for 48 h at 70°C to calculate their dry weight (DW). RWC during the desiccation period was also calculated based on the following equation according to an earlier study [9].

\text{RWC}\hspace{.5em}\text{(\%)}=(\text{FW}-\text{DW})/(\text{TW}-\text{DW})\times 100]

### 2.6 Statistical analysis

The factorial experiment with two factors (ventilation and sucrose treatment) based on a completely randomized design (CRD) was used in this study. The mean comparison for sucrose treatment at all three levels of 10, 15, and 30 mL^*−*1^ showed no significant difference from one another. Since the effect of different rates of sucrose (10, 15, and 30 g L^*−*1^) was not significant for any of the measured traits, these treatment levels were considered as extra repetitions for each of the ventilation treatments. The sucrose control level (no sucrose application) was set aside from the final analyses. Finally, the obtained data for all measured traits were subjected to one-way analysis of variance based on CRD and multiple mean comparisons using Duncan’s test (honestly significant difference [HSD]; *P* ≤ 0.05). The data analysis was performed in SAS 9.4 by means of Proc GLM, a general linear model. Linear regression [14] between *F*_v_/*F*_m_ as an independent variable and root length as the response variable in addition to the linear relationship between leaf desiccation (independent) and measured RWC (dependent) were carried out by proc reg in SAS and depicted in Excel 2019 software.

## 3 Results

### 3.1 Vegetative growth features

Ventilation and sucrose concentration variably affected the *in vitro* plant vegetative growth parameters in both Qazvini and UCB1 rootstocks. Based on the results, vegetative growth parameters were increased by ventilation (FV treatment). Pistachio plants grown in a culture media containing different concentrations of sugar (0, 10, 15, and 30 g L^*−*1^) did not show significant differences in proliferation (in both species), shoot height (in Qazvini), and leaf number (in UCB1; Table 1). Moreover, the interaction effects of ventilation with sucrose concentration were significant for both species in all growth parameters. The maximum rootstock proliferation was seen in FV treatment and midrange concentration of sugar (10 and 15 g L^*−*1^), whereas the minimum of this parameter was observed in NV treatment and sucrose-free treatment. The maximum mean values of shoot height and root length were obtained in FV treatment with either low concentration or no application of sucrose. Also, leaf number was higher in ventilated vessels with sucrose (15 g L^*−*1^) for Qazvini rootstock, which was significantly different from all other treatments. For UCB1 rootstock, however, the maximum leaf number was obtained in full ventilated sugar-free media (Table 1).

**Table 1:** Multiple mean comparison for some growth-related features in pistachio cv. Qazvini and UCB1 under *in vitro* cultivation

Ventilation	Sucrose (g L^*−*1^)	Proliferation		Shoot height (mm)		Root length (mm)		Leaf number	
		Qazvini	UCB1	Qazvini	UCB1	Qazvini	UCB1	Qazvini	UCB1
FV	0	10.75^ab^ ± 0.54	13.15^ab^ ± 0.66	39.12^a^ ± 1.96	33.14^a^ ± 1.66	4.57^a^ ± 0.23	3.76^a^ ± 0.19	17.2^abc^ ± 0.86	18.6^ab^ ± 0.93
	10	11.14^ab^ ± 0.56	13.55^a^ ± 0.68	38^abc^ ± 1.9	33.09^a^ ± 1.65	4.5^a^ ± 0.23	3.77^a^ ± 0.19	17.4^ab^ ± 0.87	18.89^a^ ± 0.94
	15	11.88^a^ ± 0.59	12.54^ab^ ± 0.63	38.61^ab^ ± 1.93	33.02^a^ ± 1.65	4.18^b^ ± 0.21	3.7^ab^ ± 0.19	17.68^a^ ± 0.88	18.88^a^ ± 0.94
	30	10.75^ab^ ± 0.54	12.57^ab^ ± 0.63	37.91^bc^ ± 1.9	32.93^a^ ± 1.65	4.07^bc^ ± 0.2	3.44^cd^ ± 0.17	17.1^abc^ ± 0.86	17.9^ab^c ± 0.9
HV	0	9.9^bc^ ± 0.5	12.2^abc^ ± 0.61	36.69^d^ ± 1.83	33.03^a^ ± 1.65	4.45^a^ ± 0.22	3.27^d,e^ ± 0.16	16.1^d^ ± 0.81	16.9^cd^ ± 0.85
	10	10.36^b^ ± 0.52	12.54^ab^ ± 0.63	37.43^bc^ ± 1.87	32.92^a^ ± 1.65	4^bcd^ ± 0.2	3.61^abc^ ± 0.18	16.6^abc^ ± 0.83	16.9^cd^ ± 0.85
	15	11.14^ab^ ± 0.56	12.2^abc^ ± 0.61	36.79^d^ ± 1.84	32.90^a^ ± 1.65	3.8^cde^ ± 0.19	3.5^bcd^ ± 0.18	17.4^ab^ ± 0.87	17.43^c^ ± 0.87
	30	9.9^bc^ ± 0.5	11.4^bcd^ ± 0.57	36.49^d^ ± 1.82	31.83^b^ ± 1.59	3.8^def^ ± 0.19	2.98^fg^ ± 0.15	16.4^bc^ ± 0.82	17.6^bc^ ± 0.88
No ventilation	0	7.53^d^ ± 0.38	9.9^d^ ± 0.5	33.16^e^ ± 1.66	30.44^c^ ± 1.52	3.8^cde^ ± 0.19	3.27^de^ ± 0.16	13.5^d^ ± 0.68	14.47^e^ ± 0.72
	10	7.53^d^ ± 0.38	11.4^bcd^ ± 0.57	33.42^e^ ± 1.67	30.52^c^ ± 1.53	3.71^de^ ± 0.19	2.99^fg^ ± 0.15	14.7^d^ ± 0.74	15.03^e^ ± 0.75
	15	8.59^cd^ ± 0.43	10.75^cd^ ± 0.54	33.33^e^ ± 1.67	30.49^c^ ± 1.52	3.69^ef^ ± 0.18	3.2^ef^ ± 0.16	16.1^d^ ± 0.81	16.14^d^ ± 0.81
	30	8.59^cd^ ± 0.43	10.75^cd^ ± 0.54	33.63^e^ ± 1.68	29.75^d^ ± 1.49	3.67^f^ ± 0.18	2.7^g^ ± 0.14	16.3^bc^ ± 0.82	16.14^d^ ± 0.81
Ventilation		**	**	**	**	**	**	**	**
Sucrose		n.s.	n.s.	n.s.	**	**	**	**	n.s.
Interaction		**	**	**	**	**	**	**	**

Means with the same superscript letters are not significantly different (Duncan 5%).

### 3.2 The effects of ventilation and sucrose concentration on fluorescence emission

Dark-adapted leaves of control and ventilated *in vitro* plants exposed to different sucrose concentrations were used to measure the induction of Chl-a fluorescence. Aside from NPQ, which showed no significant difference regarding the application of either sucrose or ventilation factors, all other measured parameters in relation to Chl fluorescence showed a significant difference in response to both factors and their interaction effects (Table 2). The results showed that *F*_0_, *F*_m_, and *F*_m′_ responded positively to ventilation and sucrose application. However, their highest mean values were recorded mostly under HV treatment and middle concentration of sucrose (10 and 15 g L^*−*1^) in both cv. Qazvini and UCB1. In addition, Qazvini rootstock (Figure 1a and Table 2) showed maximum *F*_v_/*F*_m_ in FV treatment with 15 g L^*−*1^ of sucrose. The minimum *F*_v_/*F*_m_ was detected in NV treatment with 30 g L^*−*1^ of sucrose. This ratio was increased by 36% through FV treatment and decreasing sucrose concentration to 10 g L^*−*1^. *F*_v_/*F*_m_ value was also increased by ventilation and decrease in sucrose concentration; however, the effect of ventilation was more significant than sucrose concentration. In Table 2, it can be seen that there is no significant difference between FV-treated plants exposed to 15 and 30 g L^*−*1^ sucrose. For UCB1 rootstock, the results were slightly different so that FV and HV treatments with sucrose-free medium showed the highest *F*_v_/*F*_m_ values. There was also no significant difference between HV treatment in sucrose-free medium and FV treatment exposed to 10, 15, and 30 g L^*−*1^ sucrose. Therefore, it can be suggested that sucrose concentration, notably sucrose elimination in the medium, had the most significant effect on *F*_v_/*F*_m_ value. For UCB1 rootstock, *F*_v_/*F*_m_ was increased by 40% through FV treatment with sucrose-free medium compared to NV plants, which had been exposed to 30 g L^*−*1^ of sucrose (Table 2). Since the effect of neither ventilation and sucrose was significant on NPQ, multiple mean comparisons for this feature are not presented.

**Table 2:** Multiple mean comparison for fluorescents-related parameters in pistachio cv. Qazvini and UCB1 under *in vitro* cultivation

Air ventilation	Sucrose (g L^*−*1^)	*F*_0_		*F*_m_		*F*_m'_		*F*_v_		*F*_v_/*F*_m_	
		Qazvini	UCB1	Qazvini	UCB1	Qazvini	UCB1	Qazvini	UCB1	Qazvini	UCB1
FV	0	359.4^ef^ ± 17.95	371.1^ef^ ± 18.55	1536.06^d^ ± 76.8	1552.5^b^ ± 77.6	2087.4^b^ ± 104.35	2108.05^a,b^ ± 105.4	1176.6^bc^ ± 58.8	1181.3^ab^ ± 59.05	0.77^a^ ± 0.04	0.76^a^ ± 0.04
	10	468.1^de^ ± 23.4	390^def^ ± 19.5	1636^cd^ ± 81.8	1287.2^bc^ ± 64.35	2051.1^b^ ± 102.55	1593.9^bcd^ ± 79.65	1168.1^bc^ ± 58.4	896.7^cd^ ± 44.8	0.71^bc^ ± 0.04	0.69^b^ ± 0.03
	15	511^cd^ ± 25.55	329.34^f^ ± 16.45	1714^bcd^ ± 85.7	1343.9^bc^ ± 67.15	2227.92^b^ ± 111.35	1775.13^bc^ ± 88.75	1203.1^bc^ ± 60.15	1014.5^bc^ ± 50.7	0.7^bcd^ ± 0.04	0.75^a^ ± 0.04
	30	308.56^f^ ± 15.4	597.93^a^ ± 29.85	1004.8^e^ ± 50.2	1857.2^a^ ± 92.85	1358.1^c^ ± 67.9	2515.09^a^ ± 125.75	696.28^d^ ± 34.8	1259.28^a^ ± 62.95	0.69^bcd^ ± 0.03	0.68^bc^ ± 0.03
HV	0	446.4^de^ ± 22.3	432.8^cde^ ± 21.6	1689^bcd^ ± 84.45	1366.4^bc^ ± 68.3	2122.4^b^ ± 106.1	1723.96^bc^ ± 86.15	1243^ab^ ± 62.15	933.59^cd^ ± 46.65	0.73^ab^ ± 0.04	0.68^bc^ ± 0.03
	10	765.85^a^ ± 38.25	395.5^def^ ± 19.75	2199.87^a^ ± 109.95	1236.18^c^ ± 61.8	2766.2^a^ ± 13.8	1604.1^bcd^ ± 80.2	1434.02^a^ ± 71.7	840.62^cd^ ± 42	0.65^de^ ± 0.03	0.68^bc^ ± 0.03
	15	371.8^ef^ ± 18.55	502.03^bc^ ± 25.1	1114.7^e^ ± 55.7	1378.3^bc^ ± 68.9	1501.8^c^ ± 75.05	1857.63^bc^ ± 92.85	742.91^d^ ± 37.1	876.3^cd^ ± 43.8	0.67^cde^ ± 0.03	0.63^cd^ ± 0.03
	30	805.53^a^ ± 40.25	560.1^ab^ ± 28	1879^bc^ ± 93.95	1361.6^bc^ ± 68.05	2346.9^ab^ ± 117.3	1707.54^bc^ ± 85.35	1073.4^bc^ ± 53.65	801.54^cd^ ± 40.05	0.57^f^ ± 0.03	0.59^d^ ± 0.03
No ventilation	0	752.65^a^ ± 37.6	363.6^ef^ ± 18.15	1966.2^ab^ ± 98.3	1179.22^c^ ± 58.95	2496.7^ab^ ± 124.8	1595.6^bcd^ ± 79.75	1213.6^bc^ ± 60.65	815.6^cd^ ± 40.75	0.62^ef^ ± 0.03	0.68^bc^ ± 0.03
	10	687.3^ab^ ± 34.35	472^bcd^ ± 23.6	1699^bcd^ ± 84.95	1296.3^bc^ ± 64.8	2292.2^ab^ ± 114.6	1755.32^bc^ ± 87.75	1012.47^c^ ± 50.6	823.4^cd^ ± 41.15	0.6^f^ ± 0.03	0.63^cd^ ± 0.03
	15	497.52^d^ ± 24.85	468^bcd^ ± 23.4	991.18^e^ ± 49.55	1190.05^c^ ± 59.5	1236.49^c^ ± 61.8	1491.91^cd^ ± 74.55	493.66^e^ ± 24.65	721.4^d^ ± 36.05	0.5^g^ ± 0.03	0.61^d^ ± 0.03
	30	625.4^bc^ ± 31.25	417^c–f^ ± 20.85	1224.41^e^ ± 61.2	813.21^d^ ± 40.65	1558.65^c^ ± 77.9	1095.55^d^ ± 54.75	598.99^de^ ± 29.9	395.73^e^ ± 19.75	0.48^g^ ± 0.02	0.49^e^ ± 0.02
Ventilation		**	**	**	**	**	**	**	**	**	**
Sucrose		**	**	**	**	**	**	**	**	**	**
Interaction		**	**	**	**	**	**	**	**	**	**

Means with the same letters are not significantly different (Duncan 5%).

**Figure 3:**
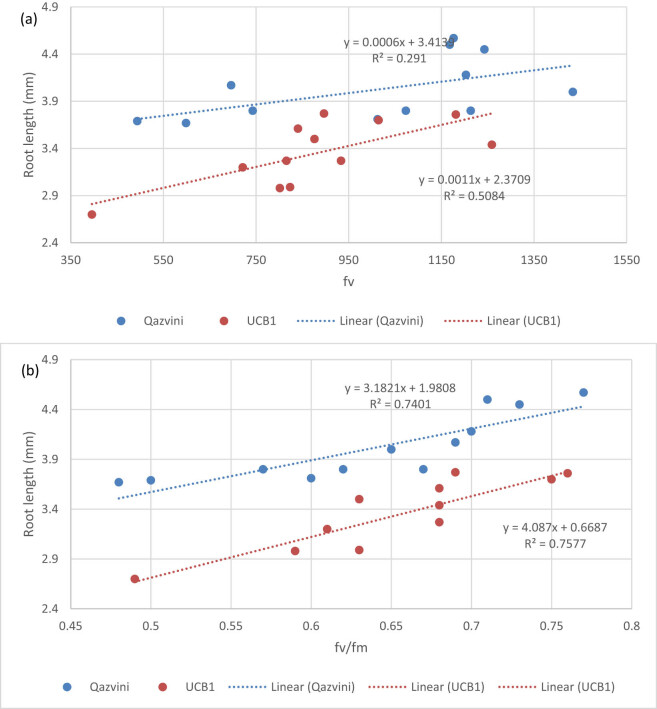
Scatter plot of root vs *F*_v_ (a) and *F*_v_/*F*_m_ (b) in pistachio cv. Qazvini and UCB1 under *in vitro* cultivation.

The scatter plots of all fluorescence feature vs root length, as response variables, were prepared, and the results showed that only *F*_v_/*F*_m_ has a significant relationship with root length (Figure 3; *r* ^2^ >0.74). The root length of both cv. Qazvini and UCB1 responded positively to the increase in the amount of *F*_v_/*F*_m_ with a linear model (Figure 3b). Although cv. UCB1 showed a higher slope than cv. Qazvini, the difference between the slopes of these two cultivars was not significant according to *t*-student test (*P* > 0.05).

### 3.3 Stomatal cell density

Morphologically, stomatal traits were completely different between the leaves of *in vitro* pistachio plants in different ventilation treatments as well as those plants in greenhouse conditions. Totally, the stomatal cell density in tissue-cultured plants was higher than that in GP, but in Qazvini rootstocks, there was a significant difference between plants treated in a greenhouse and tissue-cultured FV conditions (Table 3). Moreover, our results indicated a significant difference between different ventilation treatments so that the stomatal density of Qazvini rootstock decreased by half in FV treatment compared with NV ones. Also, the stomatal density of full ventilated UCB1 rootstocks was 54% less than NV ones. For HV treatment, stomatal density was intermediate for both UCB1 and Qazvin. Furthermore, the stomatal density of Qazvini rootstock grown in greenhouse conditions was 60% less than NV treatment. However, for UCB1, this percentage was equal to 66%. Moreover, the stomatal density of UCB1 rootstocks was more than that of Qazvini rootstocks, both in a greenhouse and *in vitro* conditions (Table 3).

**Table 3:** Stomatal and epidermal cell properties in leaves of two *in vitro* pistachio rootstocks Qazvini and UCB1

Ventilation level	Stomatal density (no mm^−2^)		Stomatal index		Epidermal cell density (no mm^−2^)		Stomatal Length (µm)		Stomatal width (µm)	
	Qazvini	UCB1	Qazvini	UCB1	Qazvini	UCB1	Qazvini	UCB1	Qazvini	UCB1
FV	115^bc^ ± 5.75	145^c^ ± 7.25	8.25^bc^ ± 0.41	8.25^c^ ± 0.41	1244.8^bc^ ± 62.2	2516.9^ab^ ± 125.8	13.88^a^ ± 0.69	12.02^b^ ± 0.6	10.89^a^ ± 0.54	9.93^ab^ ± 0.5
HV	162.5^b^ ± 8.1	277.5^b^ ± 13.85	9.47^ab^ ± 0.47	8.77^b^ ± 0.44	1529.3^b^ ± 76.45	2697.1^ab^ ± 134.85	12.76^a^ ± 0.64	11.72^b^ ± 0.59	10.72^a^ ± 0.54	9.12^bc^ ± 0.46
No ventilation	230^a^ ± 11.5	320^a^ ± 16	10.75^a^ ± 0.54	10.62^a^ ± 0.53	1885.8^a^ ± 94.25	2881.8^a^ ± 144.05	13.84^a^ ± 0.69	14.16^a^ ± 0.71	11.13^a^ ± 0.56	10.61^a^ ± 0.53
Greenhouse plant	90.75^c^ ± 4.54	107.5^d^ ± 5.35	7.2^c^ ± 0.36	4.55^c^ ± 0.23	1172.3^c^ ± 58.6	2247.2^b^ ± 112.35	14.27^a^ ± 0.71	11.53^b^ ± 0.58	10.44^a^ ± 0.52	8.36^c^ ± 0.42
Significance level	**	**	**	**	**	*	n.s.	*	n.s.	**

Means with the same letters are not significantly different (Duncan 5%).

### 3.4 Stomatal index

The present study also showed that ventilation treatments had significant effects on the stomatal index in pistachio *in vitro* plants. For Qazvini rootstocks, the stomatal index in NV treatment was almost 23% higher than FV treatment, whereas there was no significant difference between FV and HV treatments (Table 3). In UCB1, however, the stomatal index in NV treatment was almost twofold higher than FV treatment. Similar to the stomatal density, the stomatal index was significantly more in tissue-cultured plants than those grown in greenhouse conditions; however, there was no significant difference between greenhouse and tissue-cultured FV plants in both Qazvini and UCB1 species. Totally, stomatal index in Qazvini was higher than UCB1 (Table 3).

### 3.5 Epidermal cell density

The epidermal cell density of *in vitro* pistachio leaves was considerably higher than GP, but there was no significant difference between greenhouse and tissue-cultured FV treatments in both Qazvini and UCB1 plants (Table 3). Additionally, the epidermal cell density of Qazvini plants under NV treatment was 33% more than FV treatment and 37% more than GP. These values were 12 and 22% in UCB1. Moreover, the epidermal cell density of UCB1 rootstock was more than Qazvini, both in the greenhouse and *in vitro* conditions (Table 3).

### 3.6 Stomatal width and length

Based on the results, there were no significant differences in stomatal width and length of different ventilation treatments as well as greenhouse Qazvini plants (Table 3). In the case of UCB1, stomatal width and length in a tissue-cultured NV treatment were higher than other treatments, so that stomatal length in NV treatment was 15% higher than FV treatment and 18% higher than GP (Table 3). Similarly, stomatal width was 6% higher in NV treatment than FV treatment and 21% higher than greenhouse ones. Besides, the stomatal length-to-width ratio in GP was the same as that of *in vitro* plants treated with different ventilation conditions.

### 3.7 Stomatal responses to desiccation

**Figure 4:**
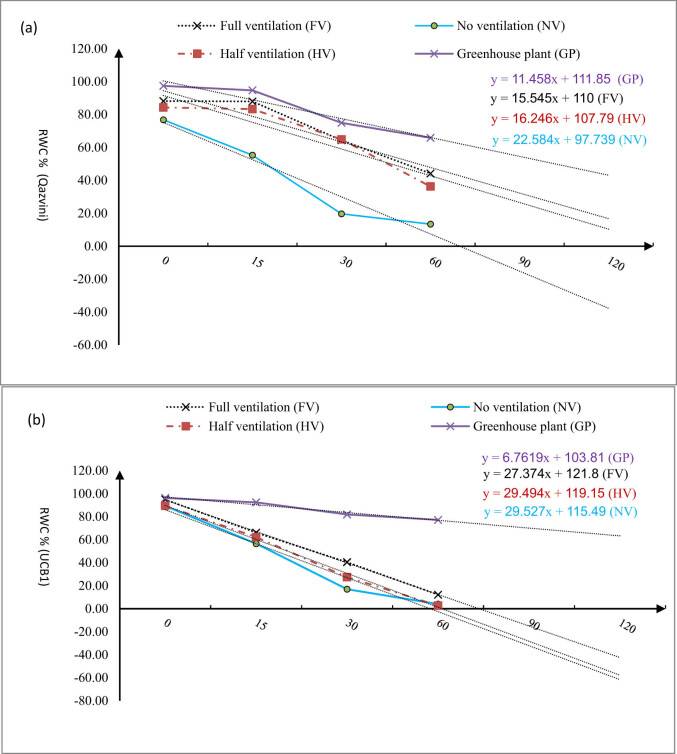
RWC% changing, as result of leaf desiccation in the Qazvini pistachio rootstock, affected by different ventilation levels in the *in vitro* conditions vs greenhouse plant in the first 60 min for cv. Qzvinig (a) and UCB1 (b), horizontal axis consists of different experimental times, vertical: RWC% changing.

By desiccation, RWC was decreased in all *in vitro* plants with different ventilation levels for both species. Regarding *in vitro* pistachio plants, RWC was sharply decreased as a result of leaf desiccation, especially in NV plantlets in the first 60 min, whereas this decrease was less strong for GP (Figure 4). For Qazvini plants, the slope of the RWC curve in NV treatment was equal to 22.58, which was 28, 31, and 49% steeper than HV, FV, and GP, respectively. As shown in Figure 4a, the slope of the RWC curve in FV treatment is closer to GP. For UCB1, on the other side, the slope of the RWC curve in NV treatment was equal to 29.52, which was 0.11, 7.2, and 69% steeper than the slope of the RWC curve HV treatment, FV treatment, and GP (Figure 4b). Although the slope of RWC curve for GP was less steep than that of *in vitro* plants, the slope of RWC curve in FV treatment was closer to the GP.

## 4 Discussion

Although the effect of ventilation and controlling the amount of photosynthetic and respirational gasses and RH of the cultured environments for the plants under *in vitro* conditions are among significant and effective factors, the effect of ventilation on the quality and quantity production of pistachio plantlets has never been assessed, to the best of our knowledge. The results of this study clearly showed that using ventilation using microfilters that can absorb microsubstances is a proper method to increase the growth of pistachio plantlets, especially the root growth, under *in vitro* culture. The shoot and root length of the pistachio plantlets have been achieved under the FV treatment and application of low to no sucrose in the media.

Several researches have provided evidence that micropropagated shoots grown in jars with high RH have shown many abnormalities, such as stomata malfunctioning with wide pore aperture [12,13]. It has also been demonstrated that exposing the vessels to high RH conditions can induce stomatal defects [14]. Based on our results, the highest stomatal density, stomatal index, and epidermal cell density were obtained in tissue-cultured unventilated plants. Furthermore, the current study results revealed that with ventilation stomata cell parameters showed more similarity to GP, and there was no significant difference between FV plants and greenhouse ones. The same results have also been reported [15]. In the present study, the stomatal parameters were less affected by *in vitro* conditions in FV vessels compared to GP. Additionally, it has been shown that *in vitro* plants’ stomatal function and their responses to closing have decreased [16]. Under the ventilation condition, the fresh air can come into the vessels and provide required sources, humidity, oxygen, and carbon dioxide to the pistachio plants. By increasing the availability of such sources, the plants can produce more vigorous roots to provide nutrients for themselves and improve their growth. In addition, such conditions would require higher photosynthesis. Therefore, the plants are inclined to produce more Chl and increase their photosynthesis rate and yield. However, different hormones affect the ability of plants to respond to the conditions and change their growth quantity. In close vessel condition, control condition with no ventilation, low evapo-transpiration rate would result in less plant ability to produce abscisic acid (ABA) phytohormone, leading to less growth and photosynthetic ability of control plants in comparison with ventilated plants. Moreover, because of high RH in closed *in vitro* vessels (more than 95%), the *in vitro* plants have never been exposed to evapo-transpiration condition to induce synthesis of ABA [17]. The ABA impact on pistachio plants is probably related to the role of ABA in retention and water content handling by the plants. Previous studies have shown that ABA can effectively alter the water condition and the plants’ growth quantity and quality; for example, a positive relationship has also been observed between leaf ABA level and the ability to conserve its water content during desiccation [4]. In agreement with our findings, in an earlier study [7] study, RWC was sharply decreased due to leaf desiccation in pistachio plants, whereas this decrease was smaller in GP. We also observed that the slope of the RWC curve was less steep for FV treated and GP compared with NV treatment. For UCB1, the slope of the RWC curve for GP was less steep than the slope of *in vitro* plants. However, the slope of RWC curve in FV treatment was closer to GP. Masle et al. [7] also noticed that low stomatal density has a critical determinant for high water-use efficiency. Consistent with our results, another study [8] stated that stomatal density and their closing mechanism in response to environmental abnormalities could tightly control plant water loss, especially in tissue-cultured plants. This can be related to more ABA biosynthesis in FV plants than NV ones and the ability of FV plants to conserve their water content during desiccation. Moreover, exposing pistachio plantlets to ventilation can induce accumulation of more ABA in the leaves and thereby causing a better stomatal closure [18]. However, reduction of photosynthetic capacity for plants can be caused by stomatal defects induced by high RH, or disorder in photosystem II. The first scenario (stomatal defects) has already been discussed in detail. To confirm the second scenario (disorder in photosystem II), we found that maximum amount of (*F*_v_/*F*_m_) was achieved in FV and in sugar-free medium and (15 g L^*−*1^) sucrose treatments for UCB1 and Qazvini pistachio rootstocks, respectively. It also seemed that ventilation could effectively decrease the high ethylene amount in the vessels and decrease damages to photosystem II functioning. In agreement with the previously mentioned results related to higher circulation of air and CO_2_ and therefore the higher ability of the plant to trap the photon from the light source, under ventilation treatments, the photosynthesis yield was significantly increased. In addition, these results regarding the influence of ventilation on pistachio’s growth parameters, especially the root organogenesis and growth, were confirmed by the significant relationship, the linear regression model [19], between root length of both cultivars and quantum photosynthesis yield of the leaves. This result is once more verifying the positive influence of ventilation on the higher quality and quantity pistachio production under *in vitro* conditions, and it almost definitely is recommendable for being applied in such systems of pistachio productions. Furthermore, our results about the maximum quantum yield of PSII (*F*_v_/*F*_m_) are in agreement with [20] as well as [16], who revealed that ventilation treatments could increase photosynthesis. Afreen et al. [21] also supported increasing photosynthetic ability by ventilation. Conversely, decreasing photosynthetic performance at high sucrose levels is consistent with the hypothesis stating that excess sucrose could motive the downregulation of photosynthesis [22]. Moreover, based on our findings, *F*_v_/*F*_m_ was reduced under closed vessels (NV condition) exposed to high sucrose concentration. Hdider and Desjardins [23] reported a higher photosynthetic ability of strawberries with transferring them from a medium containing high sucrose level to a sugar-free media. This was also the case in the present study. Similar results have also been published [24], who verified that Chl synthesis and photosynthetic ability of tobacco tissue-cultured in media with 2% sucrose were more than plants in media containing 8% sucrose. Furthermore, according to obtained results from the current study, sucrose concentration had only a small trace on the photosynthetic performance at each ventilation level, whereas ventilation was noticed to be the main effective factor. Similar results have been stated in a previous study [10] regarding walnut. Our findings are in contrast with that of an earlier study [25], who reported no significant difference between *Rehmannia glutinosa* plantlets grown in media with different sucrose concentrations in terms of their Chl content and photosynthesis performance. In line with our results, results of an earlier study [21] also indicated that somatic embryos of *Coffea arabusta* had more Chl content as well as more ability to photosynthesize under photoautotrophic conditions.

## 5 Conclusion

The results of this study showed that changing the concentration of sucrose in pistachio production under *in vitro* conditions or in line with the ventilation system had no significant effect on any of the measured traits. In addition, using ventilation showed to be highly effective for increasing the growth of *in vitro* cultures plantlets of pistachio. Ventilation was able to increase the pistachio plantlet’s root length, which has been one of the difficulties in pistachio *in vitro* cultivation. Furthermore, the quality related-features of pistachio plantlets increased with the application of ventilation. The results also showed that the photosynthesis yield of the pistachio planets under *in vitro* conditions is highly improved by applying the ventilation system, leading to improved pistachio production under *in vitro* conditions. Moreover, stomata cell parameters reposed positively to the ventilation and the trend in the FV system was almost the same as the trend in greenhouse grown plants. The final results of this study indicated that the application of low concentrations of sucrose, such as 10 mL^*−*1^ in our study, along with the application of FV systems, is recommended for producing high-quality and vigorous pistachio plantlets under *in vitro* cultivation systems.
